# The privatization of medical education in Brazil: trends and challenges

**DOI:** 10.1186/s12960-015-0095-2

**Published:** 2015-12-17

**Authors:** Mário C. Scheffer, Mario R. Dal Poz

**Affiliations:** Department of Preventive Medicine, Faculty of Medicine University of São Paulo (FMUSP), Av. Dr. Arnaldo, 455, 2o andar (Cerqueira César), São Paulo, 01246-903 SP Brazil; Institute of Social Medicine University of the State of Rio de Janeiro (UERJ), Rio de Janeiro, RJ Brazil

**Keywords:** Human resources for health, Medical education, Undergraduate medical education, Privatization, Brazil

## Abstract

**Background:**

Like other countries, Brazil is struggling with issues related to public policies designed to influence the distribution, establishment, supply and education of doctors.

While the number of undergraduate medical schools and places available on medical schools has risen, the increase in the number of doctors in Brazil in recent decades has not benefitted the population homogeneously.

The government has expanded the medical schools at the country’s federal universities, while providing incentives for the creation of new undergraduate courses at private establishments. This article examines the trends and challenges of the privatization of medical education in Brazil.

**Methods:**

This is a descriptive, cross-sectional study based on secondary data from official government databases on medical schools and courses and institutions offering such courses in Brazil. It takes into account the year when the medical schools received authorization to initiatte the activities, where they are situated, whether they are run by a public or private entity, how many places they offer, how many students they have enrolled, and their performance according to Ministry ofEducation evaluations.

**Results:**

Brazil had 241 medical schools in 2014, offering a total of 20,340 places. The private higher education institutions are responsible for most of the enrolment of medical students nationally (54 %), especially in the southeast. However, enrolment in public institutions predominate more in the capitals than in other cities. Overal, the public medical schools performed better than the private schools in the last two National Exam of Students’ (ENADE) .

**Conclusion:**

The privatization of the teaching of medicine at undergraduate level in Brazil represents a great challenge: how to expand the number of places while assuring quality and democratic access to this form of education.

Upon seeking to understand the configuration and trends in medical education in Brazil, it is hoped that this analysis may contribute to a broader research agenda in the future.

## Background 

There is an intense debate underway in Brazil about the adoption of public policies designed to influence the distribution, establishment, supply, and education of doctors.

It is known that the geographical distribution of doctors is uneven [1], the unemployment index for the profession is low [2], there is a shortage of doctors in primary care [3] and places with high healthcare needs [4], and the managers of the public health system (Sistema Único de Saúde, SUS) have trouble hiring specialists [5].

While the number of undergraduate medical schools and places available on such schools has risen, the increase in the number of doctors in Brazil in recent decades has not benefitted the population homogeneously and has run parallel to the expansion of the Brazilian health system, which includes both private healthcare [6] and the free services offered by SUS [7]. Growing health needs and epidemiological and demographic changes have resulted in a shortage of doctors, a trend that is also seen in other parts of Latin America and other regions of the world [8].

In 2013, the More Doctors programme [9, 10] was introduced in Brazil to boost the supply of undergraduate medical courses and places for residencies. It has also effected some changes to medical education, gearing it more to primary care, and has allowed for doctors from other countries to fill any supply shortfalls.

Also in 2013, the Ministry of Education expanded the medical schools at the country’s federal universities [11] and provided incentives for the creation of new undergraduate courses at private establishments [12].

This is the backdrop for this analysis of the phenomenon of privatization in undergraduate-level medical education in Brazil.

Privatization here refers to the expansion of private higher education institutions that offer undergraduate courses in medicine. These are not run by the government but by private individuals or legal entities [13]. This trend stems from the vision of the student as a consumer and of education as a product [14] and depends on the strong appeal of the brands of private higher education institutions, competition, the exploitation of market niches in socially prestigious professions, aggressive marketing, and profit-oriented pricing policies.

This article aims to characterize the privatization of medical education in Brazil. As well as demonstrating the presence and participation of public and private sectors in the provision of undergraduate courses in medicine, the analysis also aims to examine the quantitative development and geographical distribution of medical schools and to compare the relative performance of public and private institutions.

The study does not examine the funding criteria—in Brazil, a private educational establishment can receive public funds and subsidies—or privatization in public universities nor does it investigate the way educational facilities are transferred to private organizations, the sale of education products, the charging of fees, the sale of research expertise, partnerships with private business, or the adoption of private administration principles [15].

The Lei de Diretrizes e Bases da Educação (LDB), an overarching education act passed in 1996, brought the education system closer to market rules when it created “university centres” and short higher education courses, authorized simplified selection processes to replace university entrance exams, and gave private higher education establishments autonomy to make changes to the curriculum. Since then, the expansion of higher education in Brazil has been marked by privatization [16–22].

Private education has not only reaped the benefit of the upward socio-economic mobility of the population and the need for more trained professionals to meet labour requirements but has also been boosted by tax benefits and exemptions from all three levels of government, discounts on education expenses for individual taxpayers, credits and scholarships for low-income students, and loans from the Brazilian Development Bank (BNDES) to expand networks and equipment and make fixed investments and financial restructuring. This vibrant market has become the target of multiple purchases, sales, acquisitions and mergers of private establishments, new investments throughout the country, the influx of foreign capital, flotation on the São Paulo stock market (BOVESPA), and the formation of oligopolies operating in higher education.

A similar trend to that seen in Brazil also exists in Portugal and Eastern Europe, where higher education is also the target of privatization. The idea is to encourage private establishments to offer higher education to ever more students. Private institutions generally target niche markets with an eye to financial returns and have a different profile from public universities, either because they offer education in more restricted areas of knowledge or because they have limited research and postgraduate activities [23].

In Latin America, Argentina and Mexico are two countries where the majority of university places are offered by public institutions. Meanwhile, the number of places at private institutions is on the rise in Colombia and Brazil.

In recent decades in different parts of the world, there has been a rapid expansion of the number of private medical schools, changing the face of medical education, which has traditionally been provided by government-run institutions linked to large teaching hospitals [24–26]. This trend now has global implications, influencing health and medical education policies the world over. One example of the global boom in medical education is India, which has more schools of medicine than any other country (271), 137 of which are private. In the United States, there are 62 private schools out of a total of 131. It should be noted, however, that private universities in the USA are not for profit and receive a good deal of funding in the form of donations from the community and research grants from the government. In Europe, just one of the UK’s 44 medical schools is private, the University of Buckingham Medical School. Likewise, Germany only has one private school out of a total of 35, while Spain has just two out of a total of 28. In Oceania, Australia has 19 medical faculties, only two of which are private. Most of the 56 medical schools in the Caribbean region are private, which makes them a viable alternative destination for aspiring doctors from the US and Canada. In South America, 35 of Chile’s 60 schools of medicine are private. However, in some countries, including China, France, South Africa, and Canada, medical education is only offered by public institutions.

## Methods

This is a descriptive, cross-sectional study based on secondary data from official government databases on medical schools and courses and institutions offering such courses in Brazil. It takes into account the year when the medical schools received authorization to enter activity, where they are situated, whether they are run by a public or private entity, how many places they offer, how many students they have enrolled, and their performance according to Ministry of Education evaluations.

In order to define whether an institution was public or private, the criteria of the aforementioned 1996 education act (LDB) [27] were used, which establishes two administrative categories for educational establishments: public, which are “created or incorporated, maintained, and administrated by the government”, and private, which are “maintained and administrated by private individuals or legal entities”. Public higher education institutions run by federal, state, or municipal governments can be administrated directly by the government or indirectly by public foundations or autonomous public entities. Meanwhile, private institutions are classified as follows: private, when they involve one or more individuals or legal entities; community, when they are maintained by groups of individuals or one or more legal entities, such as cooperatives of parents, teachers, and students, in which case community representatives should be members of its board of directors; religious, administrated by groups of individuals or one or more legal entity that follows a specific ideology and religious orientation; and philanthropic, which are non-profit private organizations providing educational support for low-income population groups.

The medical schools are presented in this study in two main groups, public and private, without any sub-divisions or sub-classifications. Likewise, no distinction is made between the different designations of higher education institutions that can be accredited by the Ministry of Education according to their organizational structure and academic prerogatives, namely faculties, university centres, and universities [28]. The term “medical school” or “medical course” is used here to designate autonomous structures responsible for providing undergraduate education in medicine.

Two parameters are used to describe the number of students at the public and private medical schools. The first parameter is the number of students enrolled at the establishments in activity by 2012. These data were obtained from the 2012 higher education census conducted by the Instituto Nacional de Estudos e Pesquisas Educacionais Anísio Teixeira (INEP), based on information submitted by higher education institutions. The advantage of enrolment data is that it picks up on rises and falls in the number of students throughout a course (in comparison with the number of places initially authorized for each course). These changes can be caused by transfers, the recognition of equivalence of degrees earned overseas, dropouts, authorizations for increased places, higher numbers of students benefitting from scholarships and government programmes, or even reductions in places, normally temporarily, as a punitive measure after Ministry of Education inspections. However, census data on enrolments do not pick up on recent increases in the number of places available and the considerable number of new schools that have sprung up, many of which had not begun their activities when the census was taken but which could have an impact on the near future of undergraduate level medical education in the country.

In view of this limitation, a second parameter was adopted, which considers the number of places made available, authorized and increased at all medical schools and courses accredited by June 2014. In this case, the source of data was the Ministry of Education’s e-MEC online platform (http://emec.mec.gov.br/), where the processes that regulate higher education in Brazil can be monitored. Newly accredited, reaccredited, or renewed medical courses and the addition of places at existing schools are all registered on the platform. Changes in the number of courses available can be monitored by accessing the database of schools in activity and places authorized, giving an up-to-date picture of the number of graduates.

To compare the performance of public and private medical schools, the study considered the grades obtained in the National Exam of Students’ Performance (Exame Nacional de Desempenho de Estudantes, ENADE) which is used by the Ministry of Education to evaluate higher education every 3 years.

In the ENADE exam, each school is scored from 1 to 5 based on the scores of their students upon graduating.

In this study, the means and standard deviations of the ENADE scores for undergraduate courses in medicine in the last two exams (2010 and 2013) are presented. The differences between the public and private schools were tested using Student’s *t* test, and *P* values of <0.05 were considered statistically significant.

The research was approved by the Committee on Ethics in the Research of the Medical School of the University of São Paulo (# 797.424/2014).

## Results

**Fig. 1:**
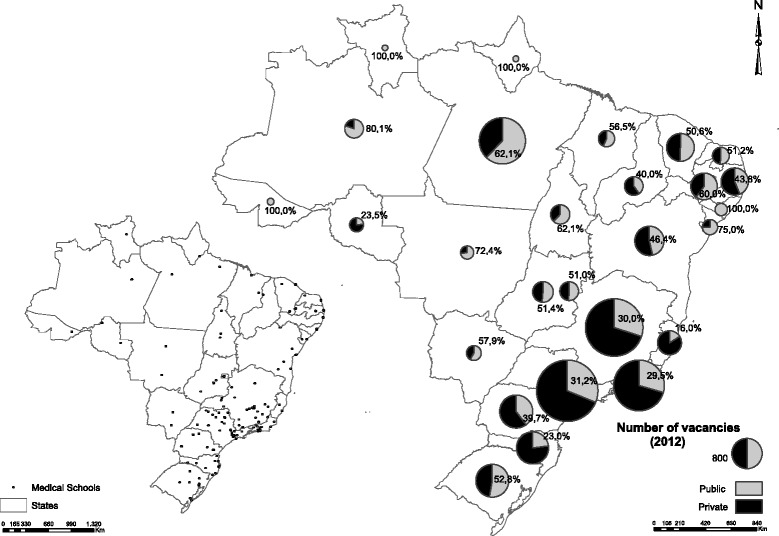
Geographical distribution of public and private medical schools and medical students per state

Private higher education institutions are responsible for most of the enrolment of medical students in Brazil, especially in the southeast (Fig. 1). However, enrolment in public institutions predominate more in the capitals than in other cities.

In 2012, there were 183 medical schools in activity in the country, with a total of 110 649 students enrolled in all 6 years of the undergraduate course, according to data from the Census of Higher Education of INEP. Of this total, 68 243 students (61.68 %) were enrolled in 109 private institutions, while 42 406 (38.32 %) in the 74 public institutions.

The southeast accounted for half of all enrolments (55 888 students, or 50.51 % of the total in the country), but the majority (71.84 %) were at private medical schools. In the state of São Paulo, 68.8 % of students were enrolled at private institutions, while in Espírito Santo, this figure reached 84.0 %. The percentage of private enrolments was lower in the north of the country (38.68 % of the 7071 students enrolled), followed by the northeast (48.50 % of 22 553 students), central west region (54.82 % of 9186 students), and south (58.82 % of 15 951 students).

There was no student enrolled at private institutions in four states of Brazil: Acre, Amapá, Roraima, and Alagoas. In the first three, there was just one public federal medical school in each state, while in Alagoas, there was one federal medical school and one state medical school.

There were 57 844 students enrolled at medical schools in the 26 state capitals and the federal district, representing 52.28 % of all enrolments, of which 53.42 % were at private institutions. The other 52 805 students, representing 47.72 % of all enrolments in medical schools, were enrolled in other cities, 70.72 % of which, enrolled at private institutions.

Brazil had 241 medical schools in 2014, offering a total of 20 340 places—a figure that includes institutions in activity or authorized to operate by July 2014 and even not yet in operation (Table 1). The majority of these places were offered at the 136 private institutions: 11 054 (54.35 %), as against 9286 places (45.65 %) at the 105 public institutions. In the north of the country, more places were available at public institutions than at private ones while the opposite was true in the south and southeast. The central west region had a fairly equal balance. The southeast, with 70 private schools, was the region with the largest number of seats available in private medical schools: 5518 (49.92 %).

**Table 1:** Medical students and number of places at public and private higher education institutions per state. Brazil, 2014

Regions	States	Public		Private		Brazil	
		No. of schools	No. of places	No. of schools	No. of places	Total schools	Total places
North	AC	1	40	0	0	1	40
	AM	2	242	1	60	3	302
	AP	1	30	0	0	1	30
	PA	4	330	2	200	6	530
	RO	1	40	3	130	4	170
	RR	1	28	0	0	1	28
	TO	2	200	2	122	4	322
	*Subtotal*	12	910	8	512	20	1422
Northeast	AL	2	130	2	200	4	330
	BA	10	568	4	500	14	1068
	CE	4	340	4	432	8	772
	MA	4	290	1	100	5	390
	PB	3	310	6	520	9	830
	PE	5	430	4	420	9	850
	PI	3	160	2	180	5	340
	RN	3	166	1	120	4	286
	SE	2	150	1	50	3	200
	*Subtotal*	36	2544	25	2522	61	5066
Southeast	ES	1	80	4	420	5	500
	MG	12	2158	24	998	36	3156
	RJ	5	660	12	1406	17	2066
	SP	11	1040	30	2694	41	3734
	*Subtotal*	29	3938	70	5518	99	9456
South	PR	7	476	8	730	15	1206
	RS	6	536	9	616	15	1152
	SC	4	236	6	294	10	530
	*Subtotal*	17	1248	23	1640	40	2888
Central west	DF	2	76	3	330	5	406
	GO	3	210	3	240	6	450
	MS	2	120	2	130	4	250
	MT	4	240	2	162	6	402
	*Subtotal*	11	646	10	862	21	1508
Brazil		105	9286	136	11 054	241	20 340

Source: Own table based on e-MEC data

Though the region with more public medical schools was the northeast (36), it is the southeast, where the private sector predominates, that offers more undergraduate places at public medical schools: 3938 places at 29 institutions. São Paulo, Rio de Janeiro, and Minas Gerais have many public institutions that offer more places and have more enrolments per year than the private institutions or even some public institutions opened more recently. The north is the region where there are more places available at public institutions (910) relatively to the private institutions (512), except in the state of Rondônia. In the other regions, the only states that have more places at public medical schools than private are Minas Gerais, Mato Grosso, Bahia, Pernambuco, Rio Grande do Norte, Maranhão, and Sergipe. Minas Gerais is the state that has the highest number of public institutions offering undergraduate courses in medicine (12 out of the national total of 105); these schools offer 2158 places a year, more than twice as many as their private counterparts (998).

São Paulo, with 41 medical schools, 30 of which are in private institutions, had 2694 places in private institutions—the largest number in the country—2.59 times higher than public institutions. Together, the four southeastern states (Rio de Janeiro, Sao Paulo, Minas Gerais, and Espírito Santo) had 2.4 times more institutions offering private courses in medicine (70) than public institutions (29), indicating the geographic preference of the educational private institutions.

In the last few years, the Ministers of Health and Education have formulated policies to promote changes in the medical curriculum towards primary healthcare such as National Guidelines for the Curriculum of Health Professional Education, Program for the Encouragement of Curricular Changes in Medical Courses (Promed), and the National Program for the Reorientation of Health Professional Education (Pró-Saúde). Although it is not fully known the impact of these initiatives on the skills of the students, it is clear, from the data, that there was no change in their geographical distribution.

When the first private school of medicine was opened at the Sorocaba’s campus of the Pontifical Catholic University of São Paulo in April 1950, Brazil already had 14 medical schools, all public. However, from the 1960s onwards, the undergraduate medical schools began to be privatized in a way that was consolidated in the mid-2000s, when the number of private schools exceeded the public. In the 1960s, only four of the 29 courses were private.

**Fig. 2:**
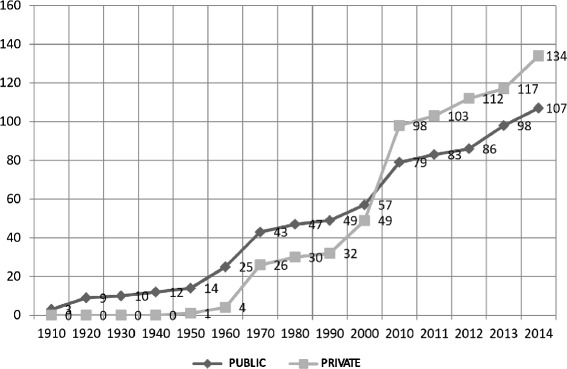
Breakdown of public and private medical schools according to year of founding. Brazil, 2014

Figure 2 shows the three emergent periods of medical schools in Brazil, indicating the steady growth of the private sector. The first dramatic increase in the number of medical schools took place between 1960 and 1979, when 50 institutions began their activities. Twenty-six of these were private, while the other 24 were public: 14 federal, eight state-run, and two municipals. Most of the courses (29) were in the southeast, while 10 were created in the south and four in the central west region. The four courses in the northeast and the three in the north were all public.

From 1980 to 1986, no new courses in medicine were created. Then a second period of growth came, from 1987 to 2007, when 93 courses were opened. The majority (65) were offered by private institutions, while the remaining 28 were public: 12 at federal institutions, 12 at state-run institutions, and four at municipal institutions. At that time, the number of private courses surpassed the public courses. From the end of 2004, of 142 medical schools in the country, over half (73) were private institutions. This trend has not changed until now. From 2008 to 2010, the pace of opening new courses decreased: only seven new courses were opened, three of which were private—four in 2008, one in 2009, and two in 2010.

The third period of marked growth in the number of medical schools began in 2011, again with private institutions taking the lead, and the data until July 2014 do not indicate any change in this trend. In these 3 1/2 years, 64 new courses were authorized, with the numbers increasing over time: nine new courses in 2011, 12 in 2012, 17 in 2013, and 26 in 2014. A total of 2958 new places were provided by these 36 private schools opened in the period, while the 28 new public courses were offering 1560 places.

The performance of the medical schools (public and private) was assessed with the last two ENADE scores available: 2010 and 2013. Each course was graded from 1 to 5, with 1 and 2 indicating poor performance.

Not all the undergraduate medical schools had ENADE scores in 2010 or 2013. The courses that did not have graduated students by 2010 or 2013 or when fewer than two students took the evaluations or were not interested in the exam (basically some estate universities) were removed from the study.

The 2010 ENADE results included 141 institutions, of which 66 were public and 75 were private. For 2013, the study included 160 institutions: 67 were public and 93 private.

**Fig. 3:**
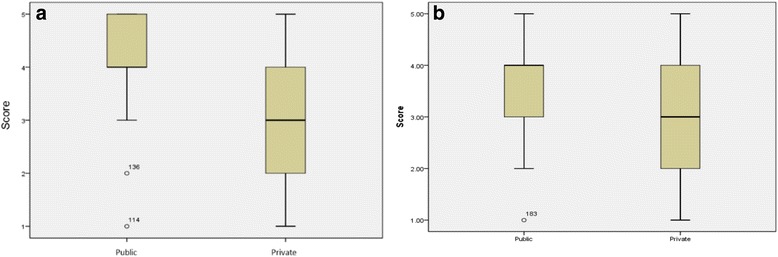
ENADE 2010 (**a**) and ENADE 2013 (**b**) scores of medical schools at public and private higher education institutions. Brazil, 2014

The performance of the public medical schools was higher than the private schools, as shown in Table 2 and Fig. 3.

**Table 2:** Comparison of ENADE 2010 and 2013 scores obtained by public and private undergraduate medical courses. Brazil, 2014

ENADE	Type of institution	Number of schools	Mean	Standard deviation	*P* value
2010	Public	66	4.170	0.82	≤0.001
	Private	75	2.960	0.97	
2013	Public	67	3.791	0.87	≤0.001
	Private	93	2.903	0.96

In 2010, 50 % of the public institutions had an ENADE score higher than 4, of a maximum of 5, reaching a high level. One public school obtained score 2 and another 1, clearly outliers of the group. As for private schools, 50 % of them were around the median and half of them below average and the other half above average.

In 2013, the performance of the public schools was below than that of the 2010 evaluation—25 % had ENADE scores less than 4. One public school obtained note 1, 25 % of them had scores between 2 and 3, and 50 % received grades between 3 and 4. The private schools’ performance in 2013 was quite similar to 2010. Although it has decreased the difference between the two groups (public and private), the performance of public schools once again outperformed the private schools.

The average statistical test (Student’s *t* test) was applied. In 2013, the average of the public schools was 3.79 (SD 0.87) while the average of the private schools was 2.90 (SD 0.96). In 2010, the average of the public schools was 4.17 (SD 0.82) while the average of private schools was 2.96 (SD 0.97).

The two evaluations, 2010 and 2013, show a statistically significant difference of means, demonstrating the best performance of public schools over private schools.

## Discussion

This study demonstrates that university-level medical education in Brazil is mostly private. Not only are more courses offered at private institutions, but they also offer more places and have more students enrolled.

We have identified some factors that could have contributed to the proliferation of private medical courses in Brazil, a phenomenon seen more clearly in three separate periods. Between 1960 and 1979, 26 private institutions began offering medical education. The grow of private education was favoured by the 1967 constitution, imposed by the military regime, which eliminated the set budget for education and supported the private sector involvement in higher education through incentives and operating licences issued by the Federal Council for Education [29].

A second period of growth identified here, from 1987 to 2007, had 65 new private medical courses opened and was associated with the new constitution passed in 1988 and the education act (LDB) passed in 1996, as well as a number of other legal measures taken by the Ministry of Education, all of which fostered a favourable regulatory environment and incentives for the growth of private higher education [30].

More recently, from 2011 to mid-2014, the opening of 36 private courses in such a short space of time was a direct response to the new federal government policies and incentives and targeted legislation, all designed to boost the offer of undergraduate education in medicine. The most influential of these measures is the More Doctors programme [9, 10] that is expanding the private undergraduate medical courses towards municipalities outside the state capitals through a competitive process [12].

Interestingly, the federal government, through the Federal University Restructuring and Expansion Programme (REUNI) and the National Policy for the Expansion of Medical Schools at Federal Higher Education Institutions [11], has significantly boosted the number of places available on medical courses at federal universities, though this has not proved enough to counterbalance the rising tide of private sector courses.

After having passed the law instituting the More Doctors programme, there has been a rising trend to privatize medical education, accompanied by a more decentralized offer of courses in smaller towns and outlying regions. This deliberate strategy of the federal government aims to provide courses to centres more distant of the traditional, allowing undergraduate medical courses to be established in municipalities with at least 70 000 inhabitants, thus containing the creation of new courses in capitals [12].

The privatization of medical education must be seen in the broader context of the expansion of private higher education in general. This market has been boosted not only by rising employment and income levels but also by government incentives for private higher education. Several mergers and acquisitions of institutions have taken place, with the formation of corporate education groups and conglomerates with footholds in many parts of the country, often linked with foreign capital and through initial public offerings on the stock market [17]. Another trend in private education in Brazil is the segmentation, either by cost (monthly fees) or by the exploitation of specialized niches [31].

One example of the private education trend in Brazil is the merger of the two largest private education companies in the country—Kroton Educacional and Anhanguera Educacional—to create one of the world’s largest education businesses [32]. Other examples of big businesses are Estácio Participações, parent company of Universidade Estácio de Sá in Rio de Janeiro, and Sistema COC de Educação e Comunicação, based in São Paulo. Meanwhile, Laureate, a global network of private academic institutions, has already acquired 12 Brazilian institutions, including Anhembi Morumbi and Faculdades Metropolitanas Unidas. These large groups have increased their market share by acquiring the smallest faculties and schools, normally in middle towns and cities, which are often in financial difficulties because they cannot compete with the fees charged by these conglomerates [17, 31].

In the case of medicine, this trend of large groups taking over the market is not yet seen. Of the 110 649 medical students enrolled in 2012, just 3.84 % were at institutions controlled by the biggest groups: Laureate (1485), Estácio (1289), Kroton (915), and Anhanguera (564). In the case of the Estácio Group, this study has not picked up the potential rise in student numbers since this institution was only recently chosen by the Ministry of Education, through its Assisted Transfer Program, to take on the students from two banned universities: Universidade Gama Filho and UniverCidade.

According to data gathered in this study, beyond the Pontifical Catholic Universities and their related institutions, which accounted for 3087 students enrolled in 2012, most of all other private institutions with medicine courses are independently owned, and many are family-owned, situated in small and middle towns and cities, away from the main metropolitan areas. In other words, the private market for medical education is still very fragmented, but this does not preclude future changes.

The limitations of this study are inherent to the secondary databases used: the time lag between the time of the study (2014) and the data used, which is the case of the 2012 INEP higher education census, while the ENADE evaluation was from 2010 and 2013, and also the different timeframes and quantitative differences between the two databases used, which in the case of e-MEC was for places available, while the census was for students enrolled.

When comparing the performance of public and private medical schools, this study found that the results of the Ministry of Education evaluations, which consider both the skills acquired by students (through exams) and the performance of the school (faculty qualifications and didactic and educational organization of the institution) [33], were sufficient to analyse the effectiveness of Brazilian higher education [34].

However, course evaluations still need to be improved, so that more appropriate weights can be given to different factors, including the intellectual merit of the students admitted via such highly competitive entrance exams as there are for medicine courses. There are also institutional and regional asymmetries in the supply of higher education, which could be better analysed and taken account of in evaluation processes.

Finally, there are limitations of a broader nature. The recent restructuring and dynamics of higher education in Brazil make it hard to use conventional categories to contrast the private and public sectors [30]. The degree to which public policies influence the education market, providing an increasing supply of direct and indirect credit instruments and subsidies for private higher education establishments in general and those offering medicine in particular, and the pace of growth of the number of courses and places available thanks to state intervention and new legal frameworks are other factors that limit this study.

## Conclusions

The privatization of the teaching of medicine at the undergraduate level in Brazil represents a great challenge: how to expand the number of places while assuring quality and democratic access to this form of education. Above and beyond the issues studied here, this prompts other questions that could be investigated in future studies.

1. With the expansion of private medical education, whose quality and performance indicators fall short of public education, further study into the evaluations conducted by the Ministry of Education could be conducted, as could new evaluation procedures and mechanisms, like progress tests given by the institutions themselves, external evaluations for graduating students, the accreditation of schools, and student evaluation methods better suited to the changes in the curriculum of undergraduate courses [35].
2. The medical education system, in the throes of expansion, should be adequately supplied with educational and teaching projects, infrastructure, and resources [36]. Evaluative research could monitor or measure the implementation of the educational proposals first formulated when the schools are accredited; whether the medical schools have the minimum infrastructure necessary, including laboratories and a library; whether they are in fact integrated into the local and regional healthcare system; whether they work in conjunction with teaching hospitals or public healthcare units capable of providing residencies and practical experience for students; and whether there is an established core faculty, with experienced, highly qualified lecturers working exclusively or priority at the school.
3. There is a need to analyse any obstacles, with a view to identifying new mechanisms to democratize access to higher education [37]. In the case of medicine, even with the new courses and places on offer, the admission procedures, which for public universities involve extremely competitive entrance exams, and the high fees charged by private courses, tend to foster inequality of access because they favour students from more affluent backgrounds. As medical courses are more competitive or expensive, few students have received incentives from the University for All programme (PROUNI), the Higher Education Student Loan Fund (Fies), and specific inclusion, quota, and affirmative action programmes.
4. Although only recently some policies have been implemented, the majority of public universities (federal and estate) have already some kind of affirmative action to reserve places in the selection process for new students, mostly quotas for blacks and Indians, combined or not with quotas for students coming from secondary public schools. In 2012, the Federal Supreme Court (STF) ruled constitutional the adoption of these policies. However, according to recent reports, these policies have had low impact in areas such as engineering or medicine [38].
5. It is known that medical residency programmes have an influence on where doctors settle [39], but it would also be worthwhile analysing the relationship between the locations of undergraduate courses and where doctors end up working. The data from this study could serve as a starting point by indicating that the highest proportion of places on medical courses is in the southeast of the country (46.5 %), followed by the northeast (25 %), south (14.2 %), central west (7.4 %), and north (6.9 %). The percentages of doctors working in Brazil per region [1] are as follows: 56.05 % in the southeast and 17.15 % in the northeast, where there are more doctors than places on medical schools and courses; 14.9 % in the south; 7.64 % in the central west; and 4.26 % in the north, where there are about the same number of doctors as there are places.
6. The public policy to stimulate the creation of new medical schools and places on these schools and courses should be evaluated systematically to appraise the nature of the funding, the costs of medical education, and the quality of the education provided, as well as adjusting the growth in supply to the changes desired for the reformulated curriculum for medical education to ensure they meet the needs of the Brazilian population and healthcare system.

Upon seeking to understand the configuration and trends in medical education in Brazil, it is hoped that this analysis may contribute to a broader research agenda in the future.

## Abbreviations

BNDES: Brazilian Development Bank
BOVESPA: São Paulo stock market
e-MEC: Ministry of Education’s e-MEC online platform
ENADE: National Exam of Students’ Performance (Exame Nacional de Desempenho de Estudantes)
FIES: Higher Education Student Loan Fund
INEP: Instituto Nacional de Estudos e Pesquisas Educacionais Anísio Teixeira
LDB: Law of Education Guidelines and Bases (Lei de Diretrizes e Bases da Educação)
PROUNI: University for All programme
REUNI: Federal University Restructuring and Expansion Programme
SUS: Health Unified System (Sistema Único de Saúde)
